# Genetic and Non-Genetic Influences during Pregnancy on Infant Global and Site Specific DNA Methylation: Role for Folate Gene Variants and Vitamin B_12_

**DOI:** 10.1371/journal.pone.0033290

**Published:** 2012-03-30

**Authors:** Jill A. McKay, Alexandra Groom, Catherine Potter, Lisa J. Coneyworth, Dianne Ford, John C. Mathers, Caroline L. Relton

**Affiliations:** 1 Institute for Ageing and Health, Human Nutrition Research Centre, Newcastle University, Newcastle upon Tyne, Tyne and Wear, United Kingdom; 2 Institute of Genetic Medicine, Human Nutrition Research Centre, Newcastle University, Newcastle upon Tyne, Tyne and Wear, United Kingdom; 3 Institute for Cell and Molecular Biology, Human Nutrition Research Centre, Newcastle University, Newcastle upon Tyne, Tyne and Wear, United Kingdom; Wayne State University, United States of America

## Abstract

Inter-individual variation in patterns of DNA methylation at birth can be explained by the influence of environmental, genetic and stochastic factors. This study investigates the genetic and non-genetic determinants of variation in DNA methylation in human infants. Given its central role in provision of methyl groups for DNA methylation, this study focuses on aspects of folate metabolism. Global (LUMA) and gene specific (*IGF2, ZNT5, IGFBP3*) DNA methylation were quantified in 430 infants by Pyrosequencing®. Seven polymorphisms in 6 genes (*MTHFR, MTRR, FOLH1, CβS, RFC1, SHMT*) involved in folate absorption and metabolism were analysed in DNA from both infants and mothers. Red blood cell folate and serum vitamin B_12_ concentrations were measured as indices of vitamin status. Relationships between DNA methylation patterns and several covariates viz. sex, gestation length, maternal and infant red cell folate, maternal and infant serum vitamin B_12_, maternal age, smoking and genotype were tested. Length of gestation correlated positively with *IGF2* methylation (rho = 0.11, *p* = 0.032) and inversely with *ZNT5* methylation (rho = −0.13, *p* = 0.017). Methylation of the *IGFBP3* locus correlated inversely with infant vitamin B_12_ concentration (rho = −0.16, *p* = 0.007), whilst global DNA methylation correlated inversely with maternal vitamin B_12_ concentrations (rho = 0.18, *p* = 0.044). Analysis of common genetic variants in folate pathway genes highlighted several associations including infant *MTRR* 66G>A genotype with DNA methylation (χ^2^ = 8.82, *p* = 0.003) and maternal *MTHFR* 677C>T genotype with *IGF2* methylation (χ^2^ = 2.77, *p* = 0.006). These data support the hypothesis that both environmental and genetic factors involved in one-carbon metabolism influence DNA methylation in infants. Specifically, the findings highlight the importance of vitamin B_12_ status, infant *MTRR* genotype and maternal *MTHFR* genotype, all of which may influence the supply of methyl groups for DNA methylation. In addition, gestational length appears to be an important determinant of infant DNA methylation patterns.

## Introduction

DNA methylation is an epigenetic modification that plays an important role in regulation of gene expression [1]. It provides a potential mechanism through which the genome can ‘capture’ the effects of environmental exposures and perpetuate their influence on physiological systems over long time periods [2]. Factors known to influence methylation patterns throughout the life course include nutrition, smoking and age [3]–[5]. Evidence of considerable inter-individual variation in DNA methylation has been documented in adults [6]–[11] but the degree of inter-individual variation in DNA methylation in humans at birth, and the factors that influence these DNA methylation patterns, are poorly understood. Emerging evidence suggests that ethnicity, parental age, maternal pregestational BMI and being born small for gestational age can influence DNA methylation [12]–[15] but it is likely that many more factors modulate the infant methylome including both environmental and genetic components [16], [17].

Factors that modulate one-carbon metabolism and so influence the provision of methyl groups via S-adenosyl methionine (SAM) for DNA methylation may be particularly important [18]. For example, status with respect to folate and the other micronutrient co-factors required for SAM synthesis via one-carbon metabolism may influence DNA methylation. In intervention studies in adult women, restricted folate intake resulted in reduced genome-wide DNA methylation [19], [20]. In addition, genome-wide DNA methylation in cord blood DNA correlated inversely with maternal plasma homocysteine concentration [21] and more recent data from the same group revealed an association between methylation of 289 CpG sites from fetal cord blood DNA with plasma homocysteine [22]. Finally, reduced methylation at the *IGF2* differentially methylated region, *H19 DMR*, in cord blood DNA has been associated with increased folic acid intake during pregnancy [23], and maternal peripheral blood DNA methylation at the *IGF2* locus was associated with maternal serum vitamin B_12_ levels [24]. The latter finding suggests a possible influence of maternal serum vitamin B_12_ on cord blood DNA methylation [24]. The influence of maternal one-carbon metabolism on methylation status in the offspring has been well documented in animal models [25]–[32] but as yet there are few studies demonstrating the effect of this maternal factor on infant DNA methylation in humans [33].

Recent studies of the heritability of DNA methylation patterns support the postulate that genetic factors are important determinants [7], [11], [34], [35]. In a study of adults examined at two time points more than a decade apart, some individuals lost but others gained DNA methylation [34] and patterns of change clustered in families, suggesting that genetic variation can influence methylation patterns [34]. Variants in genes encoding components of the one carbon metabolic cycle are likely to be important in influencing DNA methylation because of their potential influence on the methyl donor pool. Indeed, there is evidence that a common variant of the methylenetetrahydrofolate reductase (*MTHFR*) gene is associated with perturbed DNA methylation in a disease-free population [36] and in colorectal cancer [37], [38], but not, apparently, in other cancers [39]. Although one report suggests that maternal and infant *MTHFR* 677C>T does not affect cord blood methylation of the *SLC6A4* gene [40], the impact of this and other one carbon metabolism gene variants on the establishment of DNA methylation patterns during pregnancy or in early post-natal life in humans remains largely unknown.

Given the association between DNA methylation patterns and gene expression, it is plausible that aberrant methylation patterns at birth may predispose individuals to higher disease risk later in life via developmental programming [41]. The relationship between aberrant DNA methylation and cancer is well documented [42] and provides a paradigm for hypotheses which propose that epigenetic mechanisms mediate the link between environmental exposures and health outcomes in later life [2]. A recent study suggests that DNA methylation patterns at birth are associated with risk of childhood obesity [43], which has the potential to increase the likelihood of a wide range of metabolic and other diseases. As such, there is a need to establish the determinants of variation in DNA methylation at birth as a basis for both avoiding the establishment of aberrant methylation during development and the prediction and prevention of diseases later in life.

In summary, there is substantial inter-individual variation in DNA methylation patterns [6]–[11] that is likely to be explained by a combination of genetic and environmental exposures and by stochastic events. To date, little is known about the factors that determine variation in DNA methylation patterns at birth. The aims of this study were to investigate genetic and non-genetic determinants of variation in DNA methylation patterns in new-born infants and to assess the contribution of maternal factors, including folate and vitamin B_12_ concentrations and the genotype of enzymes involved in the one-carbon metabolism pathway.

## Materials and Methods

### Study population

Ethical approval to undertake this study was obtained from the Newcastle and North Tyneside Local Research Ethics Committee (07/Q0906/5). Written informed consent was obtained from all participating mothers recruited during pregnancy. Consent was obtained for use of their own biological samples and those of their child (including DNA) for epidemiological studies.

A nested cohort study was undertaken within the North Cumbria Community Genetics Project [44] and included 430 cord blood DNA samples (mean (± SD) gestation = 39.5 (1.4) weeks) for methylation and genotype analysis. Of these, peripheral blood DNA samples for genotype analysis were available from 201 mothers. Samples and data from this prospective, unselected, population-based cohort were collected between 1996 and 2003 at a single maternity unit in West Cumbria, UK. Mothers were recruited at their first antenatal appointment when they completed a health and lifestyle questionnaire and DNA was extracted from routine antenatal blood samples (mean (SD) gestation = 10.6 (4.3) weeks). Cord blood was collected and delivery details, birth weight, sex, gestational age, maternal smoking habits and maternal age were recorded. Maternal and infant red blood cell folate (RCF) and serum vitamin B_12_ analyses were conducted on whole blood prior to DNA extraction. Summary statistics are provided in Table 1.

**Table 1:** Baseline characteristics of the study population.

Characteristic	N	Median	25%, 75%
Males, number (%)	222/424 (52%)	-	-
Gestation, weeks	423	40.0	39.0, 40.0
Infants red cell folate, ngml	430	491.5	399.0, 602.0
Infants B_12_, pgml	413	323.0	232.0, 445.0
Mothers age at birth, years	326	28.6	23.5, 32.7
Smoked during pregnancy, number (%)	47/206 (23%)	-	-
Mothers red cell folate, ngml†	197	379.0	298.0, 512.0
Mothers B_12_, pgml†	158	283.0	226.0, 389.0

† Mothers' red cell folate and B_12_ concentrations were measured from routine antenatal blood samples (mean (SD) gestation = 10.6 (4.3) weeks).

### Genotype analysis

Seven polymorphisms in 6 genes involved in folate transport and in one carbon metabolism (*MTHFR* 677C>T (rs1801133), *MTHFR* 1298A>C (rs1801131), *MTRR* 66G>A (rs1801394), *FOLH1* 1561C>T (rs202676), *CβS* 644 bp ins, *RFC1* 80G>A (rs1051266) and *SHMT* 1420C>T (rs1979277)) were determined using standard RFLP methods as described elsewhere [45]. Polymorphisms with genotyping success rates less than 90%, with minor allele frequencies (MAF) less than 5% were removed prior to analysis. All variants were assessed for Hardy Weinberg Equilibrium (HWE). Allele and genotype frequencies are shown in Table S1.

### LUMA assay to determine global DNA methylation

The luminometric methylation assay (LUMA) protocol has been described in detail previously [46]. Briefly, 200 ng of genomic DNA was digested with *EcoRI*+*MspI* or *EcoRI*+*HpaII* in two separate 20 µl volume reactions containing 5 units of each enzyme (New England Biolabs) with 2 µl Tango buffer (Fermentas) for 4 h at 37°C. Digests were carried out in triplicate for each sample. 20 µl Pyrosequencing® annealing buffer (Qiagen) was then added to each reaction and the samples were analysed by Pyrosequencing® on a Pyromark™ MD system. The instrument was programmed to add dNTPs in the following steps; dATP, a mixture of dGTP+dCTP, dTTP and finally a mixture of dGTP+dCTP. Peak heights were calculated using the PyroMark™ 1.0 software. The *Hpa*II/*Eco*RI and *Msp*I/*Eco*RI ratios were calculated as (dGTP+dCTP)/dATP for the respective reactions. The *Hpa*II/*Msp*I, or methylation ratio was defined as (*Hpa*II/*Eco*RI)/(*Msp*I/*Eco*RI). A higher methylation ratio is indicative of less methylated DNA.

### Bisulfite Pyrosequencing® for loci-specific DNA methylation analysis

Bisulfite conversion of DNA was performed using EZ DNA Methylation Gold™ kit (Zymo Research) following the manufacturer's protocol. Briefly, 2 µg of genomic DNA was incubated with CT conversion reagent and incubated at the following temperatures; 98°C for 10 min, 64°C for 2.5 hr, held at 4°C. DNA was then transferred to a spin column, washed, desulphonated and purified, finally eluting in a 10 µl volume.

Quantitative bisulfite Pyrosequencing® was used to determine the percentage methylation at individual CpG sites within the differentially methylated region 0 (DMR0) of *IGF2* (NG_008849.1; 6098–6375) and promoters of *IGFBP3* (NT_007819.17; 45951336–45951104) and *ZNT5* (NT_006713.15; 18983340–18983714). Briefly, 0.2 µg of bisulfite treated DNA was added as a template in a PCR reaction using 12.5 µl Hot Star Taq mastermix (Qiagen), total volume 25 µl. For *ZNT5*, a nested PCR was carried out using 4 µl of a larger amplified region of *ZNT5*. All primer sequences and PCR conditions are shown in Table S2. Biotin-labelled PCR products were captured with streptavidin sepharose beads (GE Healthcare), and made single stranded using sodium hydroxide denaturation and a Pyrosequencing® Vacuum Prep Tool (Qiagen). Sequencing primers were annealed to the single stranded PCR product by heating to 80°C, followed by slow cooling. Pyrosequencing® was then carried out on a Pyromark™ MD system. Cytosine methylation was quantified using proprietary PyroQ CpG 1.0.6 software. All PCR and Pyrosequencing® reactions were carried out in duplicate.

For each assay, 0% and 100% methylated controls were prepared by carrying out a flanking PCR reaction for each gene of interest on genomic DNA to generate an unmethylated control, followed by *in vitro* methylation (*SssI* treatment) of an aliquot of the PCR product to generate a methylated control (please see Table S3 for primers and PCR conditions). These controls were used to rule out any amplification bias of primers for methylated DNA and to assess assay reproducibility using methods described previously [47]. All primer sets were found to be unbiased and assays were reproducible. Zero and 100% methylated controls were run routinely alongside samples as internal controls. CpG sites with poor success rates or extreme low/high methylation measures (mean methylation = 0%/100%) across the study population were removed before analysis.

### Vitamin status measurement

Maternal and infant RCF levels and serum vitamin B_12_ levels were measured as detailed elsewhere using an Abbott IMx ion capture assay for RCF and an immunoassay for serum vitamin B_12_ (Abbott GmBH, Germany) [45], [48].

### Data analysis

Correlation was assessed across the locus-specific CpG sites using non-parametric Spearman's rank correlation and where correlation between methylation at the CpG sites analysed within a single gene was at least modest (rho>0.6) mean percentage methylation values were also included in the analysis. Non-parametric Kruskal-Wallis and Spearman's rank correlation were used to assess associations between methylation levels and categorical (namely; infant sex, smoking status during pregnancy and genotype) and continuous exposure variables (namely; gestation, infant and maternal vitamin B_12_, infant and maternal RCF, and mothers age)respectively. For genetic analyses, methylation levels were initially compared across all three genotypes (i.e. applying no model). Subsequently, those variants demonstrating association were investigated further by applying specific genetic models (i.e. dominant, recessive and additive). Rare variants (Minor Allele Frequency (MAF) <15%) were analysed under a dominant model, in respect of the minor allele, only. Univariate and multiple linear regression analyses were subsequently performed to further examine the significant associations (e.g. check for confounding, assess relative and combined effect sizes). In addition to ordinary least squares (OLS) regression, robust regression was performed due to its ability to withstand violations of normality, heteroskedasticity and outliers given the non-parametric nature of methylation distributions and the moderate sample size available. All analyses were performed in STATA version 10 (Statacorp, College Station, TX).

## Results

### Correlation between methylation at CpG sites within the same gene

Methylation at three CpG sites was measured in the DMR of *IGF2*, at 5 CpG sites in the *IGFBP3* promoter and at 5 CpG sites in the *ZNT5* promoter. Methylation of individual CpG sites within each of the *IGF2* and *IGFBP3* loci were correlated (rho>0.60, data not shown) therefore mean methylation levels within each of these two loci were calculated and used for further analysis. *ZNT5* CpG site 1 was not reliably detected in the assay used and CpG site 4 was highly methylated (i.e. median methylation = 100%) and showed little inter-individual variation, so neither were included in further statistical analysis. Methylation of sites 2, 3 and 5 in the *ZNT5* promoter were not correlated, so these data were included in the analysis only as separate measures, and not used to calculate a mean value.

### Non-genetic determinants of methylation status

We investigated the impact of maternal and infant non-genetic factors on infant methylation patterns (Table 2 and Table 3). Females showed more methylation than males at *IGF2* site 2, and a longer period of gestation was correlated with increased methylation across the *IGF2* region. Conversely, methylation at site 3 of the *ZNT5* locus was negatively correlated with length of gestation. Infant B_12_ status was associated inversely with methylation across the *IGFBP3* locus and especially so at site 4, whereas maternal B_12_ concentration correlated inversely with infant global DNA methylation.

**Table 2:** Associations between methylation and non-genetic predictors.

			Association/Correlation†	
Non-Genetic Variable	Methylation Locus	N	Test Statistic	P-Value
**Infant**	**Infant**			
Sex, Males/Females	*IGF2* Site 2	194/180	4.80	0.029
Gestation	*IGF2* Mean	392	0.11	0.032
Infants' B_12_	*IGFBP3* Site 4	292	−0.16	0.007
Infants' B_12_	*IGFBP3* Mean	294	−0.12	0.048
Gestation	*ZNT5* Site 3	311	−0.13	0.017
**Maternal**	**Infant**			
Mothers' B_12_	Global*	121	0.18*	0.044

† Non-parametric Kruskal-Wallis test for association was performed between methylation and categorical predictor variables. Spearman's rank correlation was assessed between methylation and continuous predictor variables.

* A higher methylation ratio is indicative of less methylated DNA therefore the positive correlation reported shows that a higher maternal serum B_12_ level is associated with lower genomic DNA methylation.

**Table 3:** Univariate and multiple linear regression analysis.

				Standard	regression	analysis		Robust regression	analysis	Standardised
Model	Outcome	Predictor	N	Coef.	Standard error	P-value	R^2^	Standard error	P-value	beta coef.
Univariate	Global	Infant MTRR 66 G>A*	307	−0.035	0.014	0.009	0.022	0.013	0.007	−0.148
Univariate	Global	Maternal B12	121	1.994×10^−4^	1.049×10^−4^	0.060	0.030	1.105×10^−4^	0.074	0.172
Multipleł	Global	Infant MTRR 66 G>A*	117	−0.033	0.015	0.026	0.083	0.014	0.018	−0.206
		Maternal B12		7.620×10^−5^	7.090×10^−5^	0.285		6.280×10^−5^	0.228	0.097
		Sex†		−0.023	0.014	0.111		0.014	0.108	−0.146
		Gestation		0.002	0.006	0.761		0.007	0.784	0.028
Univariate	IGF2 Site 2	Sex	374	0.517	0.481	0.283	0.003	0.483	0.284	0.056
Univariate	IGF2 Mean	Gestation	392	0.294	0.175	0.093	0.007	0.182	0.106	0.085
Univariate	IGF2 Site 3	Infant MTRR 66 G>A*	382	−0.706	0.498	0.157	0.005	0.497	0.156	−0.076
Univariate	IGF2 Site 2	Infant CβS 644ins*	377	1.404	0.643	0.030	0.013	0.623	0.025	0.112
Univariate	IGF2 Site 1	Maternal MTHFR 677 C>T‡	154	2.081	0.694	0.003	0.056	0.651	0.002	0.236
Multipleł	IGF2 Mean	Sex†	153	0.828	0.636	0.195	0.080	0.661	0.212	0.105
		Gestation		0.422	0.272	0.122		0.288	0.144	0.124
		Infant MTRR 66 G>A*		−0.251	0.636	0.694		0.654	0.702	−0.032
		Infant CβS 644ins*		−0.977	0.890	0.274		1.004	0.332	−0.089
		Maternal MTHFR 677 C>T‡		1.286	0.478	0.008		0.460	0.006	0.214
Univariate	IGFBP3 Site 4	Infant B12	292	−0.002	0.001	0.031	0.016	0.001	0.007	−0.126
Univariate	IGFBP3 Site 4	Infant RFC1 80G>A*	302	0.676	0.380	0.076	0.011	0.319	0.035	0.102
Univariate	IGFBP3 Site 2	Maternal GCPII 1561C>T*	121	0.889	0.353	0.013	0.051	0.378	0.020	0.225
Univariate	IGFBP3 Site 3	Maternal MTRR 66 G>AΦ	117	−0.810	0.497	0.106	0.023	0.207	2.000×10^−4^	−0.150
Univariate	IGFBP3 Mean	Maternal MTRR 66 G>AΦ	117	−0.655	0.570	0.253	0.011	0.283	0.022	−0.106
Multipleł	IGFBP3 Mean	Infant B12	104	−0.003	0.001	4.000×10^−4^	0.159	0.001	0.001	−0.348
		Infant RFC1 80G>A*		0.273	0.268	0.311		0.274	0.321	0.099
		Maternal GCPII 1561C>T*		0.247	0.272	0.366		0.300	0.411	0.090
		Maternal MTRR 66 G>AΦ		−0.432	0.422	0.309		0.336	0.202	−0.096
		Sex†		−0.256	0.242	0.292		0.249	0.306	−0.101
		Gestation		−0.027	0.107	0.801		0.112	0.810	−0.024
Univariate	ZNT5 Site 3	Gestation	311	−1.374	0.635	0.031	0.015	0.635	0.031	−0.122
Univariate	ZNT5 Site 2	Infant RFC1 80G>A*	314	3.469	1.396	0.014	0.019	1.481	0.020	0.139
Univariate	ZNT5 Site 3	Maternal MTHFR 1298A>C*	132	8.290	2.579	0.002	0.074	2.682	0.002	0.271
Univariate	ZNT5 Site 5	Maternal MTRR 66 G>AΦ	104	−18.714	6.105	0.003	0.084	3.410	2.971×10^−7^	−0.290

* Dominant models were applied for these SNPs, hence coefficients reflect the difference in methylation level for carriers of the minor allele compared to major allele homozgyotes (reference group).

† Females were compared to males (reference group).

‡ Additive models were applied for these SNPs, hence coefficients reflect the difference in methylation level for each additional copy of the minor allele compared to major allele homozygotes (reference group).

Φ Recessive models were applied for these SNPs, hence coefficients reflect the difference in methylation level for minor allele homozygotes compared to carriers of the major allele (reference group).

ł Reduced numbers in multiple regression models are due to limited maternal genotype data and removal of outliers, consequently, these reduced numbers may in part account for the lack of significance seen with some predictor variables. Note also that mean methylation levels were utilized for multiple regression modelling despite not always demonstrating the strongest effect size with individual predictors. Standardised beta coefficients are obtained by first standardizing all variables to have a mean of 0 and a standard deviation of 1, they denote the increase in methylation for a standard deviation increase in the predictor variables. Multiple regression analysis was not performed for ZNT5 associations as mean methylation was not considered across this locus.

### Genetic determinants of methylation status

We investigated the impact of maternal and infant genotype on infant methylation status (Table 3 and Table 4). Those infants heterozygous for the *MTRR* 66A variant had increased global methylation and decreased *IGF2* site 2 methylation compared with both homozygous groups. Of note, however, this SNP did not conform to HWE across the infant subgroup (S Table 1). Infants carrying the minor *RFC1* 80A variant had increased methylation, following a dominant trend, at both *IGFBP3* site 4 and *ZNT5* site 2. Infant *CβS* 644ins had a low MAF so data were tested under a dominant model only and we found that carriers of the rare insertion had increased methylation at *IGF2* site 2.

**Table 4:** Associations between methylation and genetic predictors.

			AA			Aa			aa		Genotypic Model†		Additional Model‡	
Genetic Variant	Methylation Locus	N	Median	25%, 50%	N	Median	25%, 50%	N	Median	25%, 50%	Chi^2^	P-Value	Model	Test Statistic
**Infant**	**Infant**													
*MTRR* 66G>A	Global*	179	0.37*	0.32, 0.44	117	0.35*	0.30, 0.39	11	0.38*	0.35, 0.40	10.26	0.006	Dominant	8.82
*MTRR* 66G>A	*IGF2* Site 3	198	50.88	47.69, 53.24	171	49.59	47.14, 51.66	13	50.28	46.40, 55.15	7.51	0.023	Dominant	6.90
*C*β*S* 644ins	*IGF2* Site 2	317	51.84	49.55, 54.44	57	52.83	50.53, 55.49	3	50.64	49.86, 54.73	-	-	Dominant	4.26Φ
*RFC1* 80G>A	*IGFBP3* Site 4	94	6.98	6.33, 8.01	158	7.50	6.71, 8.41	50	7.56	6.48, 8.30	6.55	0.038	Dominant	6.52
*RFC1* 80G>A	*ZNT5* Site2	111	92.50	84.50, 97.00	151	95.00	90.00, 97.50	52	96.00	89.75, 97.50	8.21	0.017	Dominant	7.76
**Maternal**	**Infant**													
*MTHFR* 677C>T	*IGF2* Site 1	49	43.10	40.37, 46.45	83	45.40	41.69, 48.25	22	46.52	45.35, 48.47	9.13	0.010	Additive	3.02
*MTHFR* 677C>T	*IGF2* Site 2	51	50.46	48.37, 53.91	80	51.74	49.52, 54.38	22	54.11	51.53, 55.77	9.19	0.010	Additive	2.93
*MTHFR* 677C>T	*IGF2* Mean	52	47.67	45.23, 51.00	86	49.28	46.57, 51.46	22	50.14	48.31, 53.44	8.10	0.017	Additive	2.77
*MTHFR* 1298A>C	*ZNT5* Site3	60	92.25	75.00, 97.50	55	97.00	89.50, 99.00	17	96.00	91.50, 98.50	8.85	0.012	Dominant	8.85
*GCPII/FOLHI* 1561C>T	*IGFBP3* Site 2	83	5.71	5.24, 6.51	35	6.00	5.49, 7.52	3	6.15	5.98, 10.60	-	-	Dominant	4.70Φ
*MTRR* 66G>A	*IGFBP3* Site 1	47	4.82	3.39, 5.78	59	5.04	4.49, 6.12	9	3.70	2.91, 4.61	7.38	0.025	Recessive	5.32
*MTRR* 66G>A	*IGFBP3* Site 3	47	4.46	4.06, 4.94	60	4.53	4.13, 5.71	10	4.00	3.73, 4.26	7.21	0.027	Recessive	5.97
*MTRR* 66G>A	*IGFBP3* Site 5	45	6.16	5.46, 6.83	58	6.93	5.99, 8.39	10	6.31	5.62, 7.05	7.65	0.022	Dominant	6.53
*MTRR* 66G>A	*IGFBP3* Mean	47	5.58	5.13, 6.58	60	5.89	5.45, 7.09	10	5.36	5.19, 5.48	8.09	0.018	Recessive	3.82
*MTRR* 66G>A	*ZNT5* Site5	45	85.00	66.50, 93.50	51	76.00	66.00, 93.50	8	58.50	50.75, 63.25	10.57	0.005	Recessive	10.15

† Associations between methylation and SNP genotypes were tested initially under a genotypic model using a non-parametric Kruskal-Wallis Test, unless otherwise stated. Those showing association were tested further under dominant/recessive and additive models using Kruskal-Wallis and Trend tests, respectively.

‡ Test statistics and p-values from the most appropriate model are presented.

Φ SNP *GCPII/FOLHI* 1561C>T and *CβS* 644ins were tested under a dominant model (with respect to the minor allele) only due to their low MAF (i.e. 5–15%). *A higher methylation ratio is indicative of less methylated DNA.

Maternal genotype also influenced infant DNA methylation. The maternal minor *MTHFR* 677T variant was associated with increased infant methylation at the *IGF2* locus (site 1, site 2 and mean) following an additive model; methylation of the *ZNT5* site 3 locus was increased in infants of mothers carrying one or more copies of the minor *MTHFR* 1298C allele; infants of mothers homozygous for the minor *MTRR* 66A variant had decreased methylation at the *IGFBP3* locus (sites 1, 3 and 5 and mean methylation at this locus) compared with infants of mothers carrying the major *MTRR* 66G allele; the same recessive pattern was also observed at the *ZNT5* site 5 locus. The maternal *GCPII/FOLHI* 1561C>T SNP had a low MAF so data were tested under a dominant model and we observed that the major homozygous maternal genotype was associated with lower infant methylation at the *IGFBP3* site 2 locus compared with heterozygous and minor homozygous maternal genotypes.

Individually, genetic and non-genetic predictor variables contributed ∼0.3 to 8% of the variability in infant methylation levels, as shown by linear regression r^2^ values (Table 3). In addition, effect sizes on methylation were similar for both genetic and non-genetic factors. Furthermore, none of these univariate associations were confounded by or demonstrated evidence of interaction with sex and/or gestational length. Overall, the combination of genetic and non-genetic predictors accounted for ∼8 to 16% of the total variation in infant methylation levels. It should be noted that, these regression analyses are likely to be underpowered for a number of reasons (e.g. limited maternal genotype data, use of mean methylation as the outcome measure, and non-parametric nature of methylation data), so that the apparent lack of significance in some models should be treated with caution.

## Discussion

The determinants of DNA methylation patterns, including the involvement of folate and other micronutrient co-factors involved in one-carbon metabolism, are the focus of considerable research interest [4], [49]. This may be expected given the central role of these micronutrients in the generation of SAM - the methyl donor for DNA methylation. In addition, evidence is emerging that ageing and a wide range of environmental exposures including nutrition and smoking [3], [10], [19], [50]–[57] as well as heritable components [7], [11], [34], [35] may modulate DNA methylation patterns throughout the life-course. However, the impact of these factors, singly and in combination, on inter-individual variation in DNA methylation patterns at birth is largely unknown.

In this study we examined global and gene specific methylation patterns in infants in relation to both non-genetic and genetic factors involved in one carbon metabolism. For this purpose, we chose 3 genes with contrasting degrees of methylation; *IGF2*, an imprinted locus with mean methylation ∼50%, *IGFBP3*, constitutively methylated at low levels (∼5%), and *ZNT5*, constitutively methylated at high levels (∼90%). We chose to investigate the *IGF2* gene as it is one of the more frequently investigated loci for DNA methylation demonstrating altered methylation in response to environmental influences [11], [15], [23], [33]. Furthermore, both IGF2 and IGFBP3 are members of the IGF system, which is important for intrauterine growth [58], hence the investigation of DNA methylation at the *IGFBP3* locus. Finally we selected the *ZNT5* gene for analysis as we had previously observed inter-individual variation in methylation at this locus in DNA from human colonic mucosal biopsies (Coneyworth, Mathers & Ford, unpublished data). We observed that both non-genetic and genetic factors explained between 0.3 and 8% of the inter-individual variation in both global and gene specific DNA methylation in infants, with the combination of both factors accounting for up to 16%. We report that increased maternal serum vitamin B_12_ was indicative of lower infant global DNA methylation, and that higher infant serum vitamin B_12_ concentration was associated with reduced methylation at *IGFBP3* site 4, and across the *IGFBP3* locus. Since vitamin B_12_ is a rate-limiting co-factor for methionine synthase reductase (MTRR) in the conversion of homocysteine to methionine, an integral step in methyl group donation, altered vitamin B_12_ supply may influence DNA methylation through SAM availability. Higher vitamin B_12_ status may result in increased SAM which would increase the SAM:*S*-adenosylhomocysteine (SAM∶SAH) ratio and alter the kinetics of methyl group donation. Moreover, in the current study, variation in both maternal and infant genotype at the *MTRR* locus resulted in changes in infant methylation, providing further evidence that aberrations at this point of one carbon metabolism might affect the capacity to methylate DNA (although it is pertinent to state that larger studies will be required to definitively assess effect the relationship between the *MTRR* 66A>G variant and DNA methylation patterns, given that this SNP was not in Hardy-Weinberg equilibrium in this study). Although Wettergren *et al* (2010) [59] reported no effects of the *MTRR* 66G>A variant on *p16INK4A* hypermethylation in the mucosa of colorectal cancer patients, de Vogel *et al* (2009) observed that *MLH1* hypermethylation among female colorectal cancer cases was inversely associated with carriage of the *MTRR* 66G>A variant [60]. In the present study, both vitamin B_12_ concentrations and variation in the gene involved in vitamin B_12_ metabolism were associated with altered DNA methylation. This observation suggests that further investigation of the effects of both vitamin B_12_ and the *MTRR* 66G>A genotype on one-carbon metabolism are warranted to understand the effects of both the vitamin and SNP on DNA methylation. Both experimentally-based and mathematical modelling-based approaches could be applied to advance understanding in this area [61], [62] to account for the influence of complex interactions at multiple nodes within the one - carbon metabolic pathway on the phenotype of DNA methylation.

As noted above, folate is an important contributor of methyl groups to one-carbon metabolism and hence a major determinant of the quantity of SAM available for the methylation of DNA. Human intervention studies have shown that moderate restrictions in folate intake reduced genome-wide DNA methylation [19]–[20]. More recently an observational study found that higher genome-wide methylation in DNA from colonic mucosa was associated with higher serum and erythrocyte folate concentrations [53]. We hypothesised that RCF concentration would correlate positively with infant genome-wide DNA methylation but we found no support for this hypothesis in the present study. Previously, Fryer *et al* (2009) [21] reported no association between cord serum folate or maternal folic acid intake and infant LINE-1 DNA methylation (an index of non-coding genome-wide methylation), but observed an inverse correlation between LINE-1 methylation and homocysteine concentration in cord blood. Furthermore, methylation patterns of 289 CpG sites from fetal cord blood DNA were found to be significantly associated with plasma homocysteine, but not serum folate concentrations [22], suggesting that homocysteine, a functional indicator of availability of one-carbon supply for DNA methylation which is influenced by several micronutrients may be a better biomarker in this context than folate per se. It is therefore plausible that, despite the lack of an association between measures of folate status and DNA methylation in this study, other micronutrient co-factors in one-carbon metabolism may influence methyl group donation which is consistent with our observations relating to vitamin B_12_.

Genetic variation in the maternal *MTHFR* gene was associated with methylation levels at the *IGF2* and *ZNT5* loci and demonstrated some of the largest individual effect sizes (∼6–7%). The *MTHFR* 677C>T and 1298A>C variant were selected for investigation because they result in elevation of total plasma homocysteine and lower circulating concentrations of folate [63] and as such can be used as unconfounded proxies for high homocysteine/low folate using a Mendelian randomization approach [64]. Using this approach there was evidence for an association between maternal homocysteine/folate levels and DNA methylation in infants in the present study, inconsistent with the null relationship observed between the blood based metabolites themselves and DNA methylation. However, the present study is limited by a modest sample size which may explain these inconsistencies. The current study was limited to the analysis of seven polymorphisms and a more comprehensive appraisal of genetic variation in one-carbon metabolic pathway may uncover further associations with DNA methylation pattens.

In this study, maternal smoking did not have any discernable effect on infant DNA methylation. Previously, Breton *et al* (2009) [54] reported lower methylation at AluYb8, but not LINE-1 elements, in buccal cell DNA of children exposed to tobacco smoke prenatally as well as increases in methylation in two genes - *AXL* and *PTPRO -* out of eight loci studied. Furthermore, cord serum global DNA methylation had an inverse relationship with serum cotinine levels, indicating genomic hypomethylation in the infants exposed to smoking *in utero* [65]. Conversely, Launay *et al*. recently reported increased DNA methyltransferase activity, decreased DNA methylation and increased gene expression of the monoamine oxidase (*MOA-B*) gene in smokers, suggesting bidirectional gene specific effects of smoking on DNA methylation [66]. The lack of an association between infant DNA methylation and maternal smoking during pregnancy observed in this study may be due to a) use of a different measure of global methylation compared with previous studies and b) the specific target genes chosen in this study compared with other loci whereas methylation of other loci may be plastic in response to smoking.

We observed that DNA methylation patterns were influenced by length of gestation in a gene specific manner; *IGF2* methylation was positively correlated with gestation length whereas this correlated negatively with *ZNT5* methylation. Previous work has shown that global DNA methylation in the baboon fetus follows a tissue-specific trajectory during the second half of gestation with decreased global DNA methylation in the frontal cortex and no change in the heart during the later stages of pregnancy [67]. Furthermore, it was reported recently that prematurely born infants had lower global DNA methylation (measured as LINE-1 methylation) in cord blood compared with term infants, suggesting that changes in fetal DNA methylation are ongoing during late pregnancy [68]. In a study of effects of maternal characteristics on methylation of selected genes in umbilical cord genomic DNA, maternal BMI correlated positively (r = 0.41) with methylation of the peroxisome proliferator-activated receptor-γ co-activator 1α gene (*PPARGC1A*) but there was no significant relationship between methylation of this gene and gestational age [14]. To the best of our knowledge, ours is the first study demonstrating effects of gestational age on gene-specific DNA methylation in infant cord blood DNA, offering additional evidence for a role of gestation length in the determination of DNA methylation patterns at birth.

In conclusion, the findings of this study are consistent with the hypothesis that modulation of one-carbon metabolism influences DNA methylation in the newborn human infant. As this area of research is still in its infancy, much remains unknown about how an individual's DNA methylation profile is established during development, what factors might influence the fidelity of these profiles during the life course and, ultimately the consequences of these altered profiles for long term health and wellbeing. This study provided an opportunity to appraise the relationship between maternal genotype and some environmental exposures on DNA methylation in infants. By measuring both global and site specific DNA methylation in 3 genes, we have contributed to the limited existing data concerning infant methylation in response to genetic and environmental factors. Although a more comprehensive investigation of methylation at other loci throughout the genome would provide deeper insights into the determinants of DNA methylation patterns at birth, the findings from this study underscore the complexity of the relationship between environmental and genetic determinants and DNA methylation status. We provide evidence that variation in one-carbon metabolism by environmental and genetic factors, specifically vitamin B_12_ the *MTRR* 66G>A SNP and *MTHFR* variants can influence infant methylation. In addition, gestational length appears to be an important determinant of infant DNA methylation patterns.

## Supporting Information

Table S1 **Genotype and allele frequencies of genetic variants.**
(DOCX)

Table S2 **Primer sequences and PCR and Pyrosequencing® conditions.**
(DOCX)

Table S3 **Primer sequences and PCR conditions for flanking PCRs to generate 0 and 100% methylated controls.**
(DOCX)
