# Supplementary material for: Genetic and Non-Genetic Influences during Pregnancy on Infant Global and Site Specific DNA Methylation: Role for Folate Gene Variants and Vitamin B12
Source: PLoS One. 2012 Mar 30;7(3):e33290. doi: 10.1371/journal.pone.0033290 (PMC3316565; doi:10.1371/journal.pone.0033290)
Supplement: Table S1 — Genotype and allele frequencies of genetic variants. (DOCX) [file pone.0033290.s001.docx]

**Supporting Information Table 1.** Genotype and allele frequencies of genetic variants.

|  |  |  |  | **Genotypes, N (Freq)** | |  | |  |  |
| --- | --- | --- | --- | --- | --- | --- | --- | --- | --- |
| **Variant** | **Rs number** | **Alleles*** | **N** | **AA** | **Aa** | | **aa** | | **HWE P-Value** |
| **Infant** |  |  |  |  |  | |  | |  |
| *CβS* 644ins | - | W:I | 427/430 | 362 (0.85) | 62 (0.15) | | 3 (0.01) | | 0.741 |
| *GCPII/FOLHI* 1561C>T | rs202676 | C:T | 424/430 | 330 (0.78) | 92 (0.22) | | 2 (<0.01) | | 0.142 |
| *MTRR* 66G>A | rs1801394 | G:A | 427/430 | 219 (0.51) | 194 (0.45) | | 14 (0.03) | | <0.001 |
| *MTHFR* 677C>T | rs1801133 | C:T | 413/430 | 187 (0.45) | 177 (0.43) | | 49 (0.12) | | 0.506 |
| *MTHFR* 1298A>C | rs1801131 | A:C | 413/430 | 207 (0.50) | 167 (0.40) | | 39 (0.09) | | 0.555 |
| *RFC1* 80G>A | rs1051266 | G:A | 427/430 | 144 (0.34) | 213 (0.50) | | 70 (0.16) | | 0.618 |
| *SHMT* 1420C>T | rs1979277 | C:T | 427/430 | 200 (0.47) | 179 (0.42) | | 48 (0.11) | | 0.438 |
| **Mothers** |  |  |  |  |  | |  | |  |
| *CβS* 644ins | - | W:I | 178/201 | 146 (0.82) | 31 (0.17) | | 1 (0.01) | | 1.000 |
| *GCPII/FOLHI* 1561C>T | rs202676 | C:T | 179/201 | 122 (0.68) | 53 (0.30) | | 4 (0.02) | | 0.790 |
| *MTRR* 66G>A | rs1801394 | G:A | 175/201 | 58 (0.33) | 94 (0.54) | | 23 (0.13) | | 0.156 |
| *MTHFR* 677C>T | rs1801133 | C:T | 171/201 | 78 (0.46) | 73 (0.43) | | 20 (0.12) | | 0.729 |
| *MTHFR* 1298A>C | rs1801131 | A:C | 175/201 | 76 (0.43) | 84 (0.48) | | 15 (0.09) | | 0.301 |
| *RFC1* 80G>A | rs1051266 | G:A | 175/201 | 54 (0.31) | 90 (0.51) | | 31 (0.18) | | 0.644 |
| *SHMT* 1420C>T | rs1979277 | C:T | 170/201 | 70 (0.41) | 78 (0.46) | | 22 (0.13) | | 1.000 |

W=Wild type, I=Insertion

*Major allele : Minor allele
