# Supplementary material for: Genetic and Non-Genetic Influences during Pregnancy on Infant Global and Site Specific DNA Methylation: Role for Folate Gene Variants and Vitamin B12
Source: PLoS One. 2012 Mar 30;7(3):e33290. doi: 10.1371/journal.pone.0033290 (PMC3316565; doi:10.1371/journal.pone.0033290)
Supplement: Table S2 — Primer sequences and PCR and Pyrosequencing® conditions. (DOCX) [file pone.0033290.s002.docx]

**Supporting Information Table 2**. Primer sequences and PCR and Pyrosequencing® conditions

|  |  |  | **PCR** |  |  |  |  | **Pyrosequencing** |
| --- | --- | --- | --- | --- | --- | --- | --- | --- |
| **Gene** | Forward Primer | Reverse Primer | Primer Concentration (pmol) | Size (bp) | Annealing (°C) | Magnesium (mM) | Q solution (µl) | Sequencing Primer |
| *IGF2** | tgg ata gga gat tga gga gaa a | Biotin-aaa ccc caa caa aaa cca ct | 5 | __ | 60 | 2.5 | 2.5 | ttt ttt agg aag tat agt ta |
| *IGFBP3* | gga att aaa ttt tag aaa g | Biotin-tct aca aaa acc aaa ata t | 10 | 232 | 40 | - | 2.5 | gag ttg tat gtt agt ttt tt |
| *ZNT5* | gga gta gga gag aag gtt atg | tca ctc ccc cat aac aaa aac | 10 | 1078 | 53 | - | - | *N/A* |
| *ZNT5 Nested* | gtt tgt tga gga ggt aaa | Biotin- ctc cta acc tca aat aat c | For = 10 Rev = 8.3 | 342 | 41 | - | - | ttt ggt tgg ggg ag |

*Primer design taken from Heijmens et al 2007[11]
