# Supplementary material for: Genetic and Non-Genetic Influences during Pregnancy on Infant Global and Site Specific DNA Methylation: Role for Folate Gene Variants and Vitamin B12
Source: PLoS One. 2012 Mar 30;7(3):e33290. doi: 10.1371/journal.pone.0033290 (PMC3316565; doi:10.1371/journal.pone.0033290)
Supplement: Table S3 — Primer sequences and PCR conditions for flanking PCRs to generate 0 and 100% methylated controls. (DOCX) [file pone.0033290.s003.docx]

**Supporting Information Table 3.** Primer sequences and PCR conditions for flanking PCRs to generate 0 and 100% methylated controls

| **Gene** | Forward Primer | Reverse Primer | Primer Concentration (pmol) | Size (bp) | Annealing (°C) | Magnesium (mM) |
| --- | --- | --- | --- | --- | --- | --- |
| *IGF2* | att att tta tag gta aag t | gtt taa tag aag ggt ttc g | 10 | 585 | 60 | 2.5 |
| *IGFBP3* | caa gac ttc gcc tgc caa | cac ctg ctc ctc gtg ctc | 10 | 295 | 65 | - |
| *ZNT5* | gac aca gga acc cac att cc | cgg ctc cgg ctc act agc aca cac | 10 | 2200 | 60 | - |
